# Interprofessional Collaborations on Interventions for People With Loneliness: A Scoping Review

**DOI:** 10.1002/nop2.70239

**Published:** 2025-05-21

**Authors:** Lok Ying Chu, Youjuan Zhang, Graeme Drummond Smith, Ken Hok Man Ho

**Affiliations:** ^1^ The Nethersole School of Nursing The Chinese University of Hong Kong Hong Kong SAR China; ^2^ Department of Applied Social Sciences The Hong Kong Polytechnic University Hong Kong SAR China; ^3^ School of Health Sciences Saint Francis University Tseung Kwan O Hong Kong; ^4^ School of Nursing & Midwifery La Trobe University Melbourne Victoria Australia

## Abstract

**Aim:**

To describe the types and effects of interprofessional collaborations (IPC) interventions, the disciplines involved, the target population and the barriers and facilitators in implementation for tackling loneliness.

**Design:**

Scoping review.

**Review Methods:**

Following Arksey and O'Mally's framework, two authors screened the title, abstract and full text of the identified studies. Characteristics of included studies and IPC interventions were extracted into tables. The PAGER framework was applied for data analysis.

**Data Sources:**

Keyword search was conducted on OVID Medline, OVID PsycINFO, OVID Embase, EBSCO CINAHL and ProQuest databases from January 2003 to October 2023.

**Results:**

Eleven eligible articles were included. IPC was generally effective in alleviating loneliness among older adults. Professionals involved students of healthcare professionals, nurses, physicians, occupational therapists, physiotherapists, psychologists, pharmacists, social workers, artists and engineers. IPC interventions were delivered face‐to‐face or by digital technology. IPC interventions involved social groups, intergenerational support, social skill training, art therapy, home visiting, coordinated care pathways and digital rehabilitation. Both facilitators and barriers to implementing IPC interventions for tackling loneliness were identified.

**Conclusion:**

Interprofessional collaboration employing digital technology has been a recent trend for tackling loneliness among older adults. However, the collaborative ways between various disciplines to develop and implement IPC interventions for tackling loneliness were unclear. Nursing demonstrated relatively low engagement in IPC studies for loneliness.

**Implications for the Profession and/or Patient Care:**

Nurses should expand their roles in IPC to tackle the psycho‐social‐spiritual aspects of loneliness. Involving an intergenerational approach is important for older adults.

**Impact:**

- There is no systematic mapping and synthesis of information on IPC for tackling loneliness.
- IPC interventions are generally effective in alleviating loneliness among older adults; however, ways of collaboration between disciplines require further attention.
- Nurses across a variety of settings in the community can become actively involved in IPC interventions for loneliness.

**Reporting Method:**

PRISMA‐ScR.

No Patient or Public Contribution.

Protocol Registration Number: DOI: 10.17605/OSF.IO/PJDH2.

## 1 Introduction

Loneliness is an unpleasant experience when there is a discrepancy between one's desired and one's actual level of social relationships, either quantitatively or qualitatively (Perlman and Peplau 1981; World Health Organization 2021). Systematic review showed that levels of loneliness are prevalent across the whole lifespan around the world, ranging from 9.2% to 24.2%, including older adults (Surkalim et al. 2022). A growing body of research has linked loneliness with a variety of adverse physical (Hodgson et al. 2020; Leigh‐Hunt et al. 2017; Lim et al. 2020; Valtorta et al. 2016) and mental health outcomes (Griffin et al. 2020; Lara et al. 2019). One meta‐analytic review reported that loneliness increased all‐cause mortality by 26% (Holt‐Lunstad et al. 2015), which was comparable to obesity and physical inactivity (World Health Organisation 2021). Loneliness has emerged as a pressing interprofessional concern, demanding immediate attention and effective solutions (Wilmoth et al. 2023). As such, it is important to understand what and how interprofessional collaboration interventions are implemented for people experiencing loneliness.

## 2 Background

Loneliness is a complex issue involving psychological, social and environmental factors that contribute to its development and persistence (Hutten et al. 2022). Therefore, interprofessional collaboration (IPC) is crucial for interventions aimed at alleviating loneliness, as the complex nature of loneliness requires a multi‐dimensional approach (Franse et al. 2018). Interprofessional collaboration refers to ‘the practice of working with practitioners from different disciplines as well as the patients/family in a collaborative relationship to deliver coordinated healthcare’ (Ketcherside et al. 2017, 370). Conceptually, IPC can be understood by a continuum from multi‐disciplinarity through interdisciplinarity to transdisciplinarity (Careau et al. 2018; Vyt 2008). In multi‐disciplinarity, different healthcare workers act independently on one client. In interdisciplinarity, knowledge is exchanged and integrated by practitioners to provide care. In transdisciplinarity, team members share goal setting and a reference framework, which transcend disciplinary boundaries (Vyt 2008).

Interprofessional collaboration has long been recognised as an effective approach to promote mental health and well‐being in a variety of populations (Reeves et al. 2018). For instance, an earlier study implementing an IPC care model involving collaboration between nurses and social workers for patients with bipolar disorder demonstrated positive outcomes in terms of social functioning, quality of life, and treatment satisfaction (Bauer et al. 2006). Another study focused on information sharing between nurses and psychiatrists through monthly telephone calls and outreach services significantly reduced the manic symptoms of psychiatric patients (Simon et al. 2006). An umbrella review on IPC in primary care summarised six practical types of IPC that mainly involve the collaboration between primary care physicians, nurses and pharmacists (Rawlinson et al. 2021). However, as loneliness is seldom tackled in the primary care system (Chana et al. 2016), there is a lack of information about IPC services/interventions targeting loneliness for affected individuals in community settings.

Unlike psychiatric disorders, loneliness is an inevitable phenomenon that is encountered by all age groups across the lifespan (Surkalim et al. 2022). As a complex and multi‐faceted issue, loneliness requires professionals' comprehensive understanding of the unique challenges faced by individuals experiencing loneliness and the factors that may contribute to their conditions (World Health Organization 2021). In an integrative review of interventions to reduce social isolation and loneliness among older people, Gardiner et al. (2018) raised at least six categories of interventions for loneliness, namely social facilitation interventions, psychological therapies, health and social care provision, animal interventions, befriending interventions and leisure/skill development. However, details about how and what health and social care disciplines collaborate to deliver loneliness interventions are lacking. The inherent differences in the nature of loneliness also make it challenging to directly generalise existing IPC models in mental health problems to address loneliness. Existing understanding about the barriers (e.g., lack of time and training, role identity) and facilitators (e.g., tools to improve communication, co‐location and recognition of other professionals' skills and contribution) of IPC interventions are also limited to the primary care settings (Rawlinson et al. 2021). With scant systematic evidence, the application and effectiveness of IPC interventions in targeting loneliness remain systematically mapped and synthesised in this area.

## 3 Study Aim

To address the research gap, this scoping review aimed to obtain a comprehensive view of IPC for people experiencing loneliness.

### 3.1 Research Question

What and how are IPC interventions implemented for people experiencing loneliness?

### 3.2 Objectives

1. To describe the IPC interventions and their effects in alleviating loneliness;
2. To describe the types of collaboration and the disciplines involved in IPC interventions for alleviating loneliness;
3. To identify the population groups with loneliness served by IPC interventions; and
4. To describe the facilitators and barriers affecting the implementation of IPC interventions for people with loneliness.

## 4 Method and Analysis

### 4.1 Design

The framework of Arksey and O'Malley (2005) was employed, which included identifying research questions, identifying relevant information, study selection, charting the data, collating, summarising and reporting the results. The PAGER framework was employed to report the results systematically (Bradbury‐Jones et al. 2022). This scoping review is reported according to the PRISMA‐ScR (Tricco et al. 2018), as detailed in Appendix S1. The review protocol has been registered and can be accessed on OSF (registration number: DOI 10.17605/OSF.IO/PJDH2).

### 4.2 Search Methods

Databases, including OVID Medline, OVID PsycINFO, OVID Embase, EBSCO CINAHL and ProQuest, were searched from inception to October 2023. Key search terms were ‘loneliness’, ‘interprofessional collaboration’, ‘interprofessional’, ‘multiprofessional’, ‘transprofessional’, ‘multidisciplinary’, ‘interdisciplinary’ and ‘transdisciplinary’.

### 4.3 Inclusion and Exclusion Criteria

Articles in any language and published from January 2003 to October 2023 were included. Appendix S2 showed the full search strategy. Studies were selected according to the Population, Concept and Context framework (Peters et al. 2020). Table 1 showed the inclusion and exclusion criteria.

**TABLE 1:** Inclusion and exclusion criteria.

	Inclusion criteria	Exclusion criteria
Population	Any population with loneliness	Population only mentioning about social isolation
Concept	Collaboration between disciplines	Services or interventions only involving one discipline
Context	Any settings	Hospitalisation
Types of studies	Quantitative study, qualitative study and mixed‐methods study	Reviews, editorials, commentary articles and conference abstracts

### 4.4 Study Selection

**FIGURE 1:**
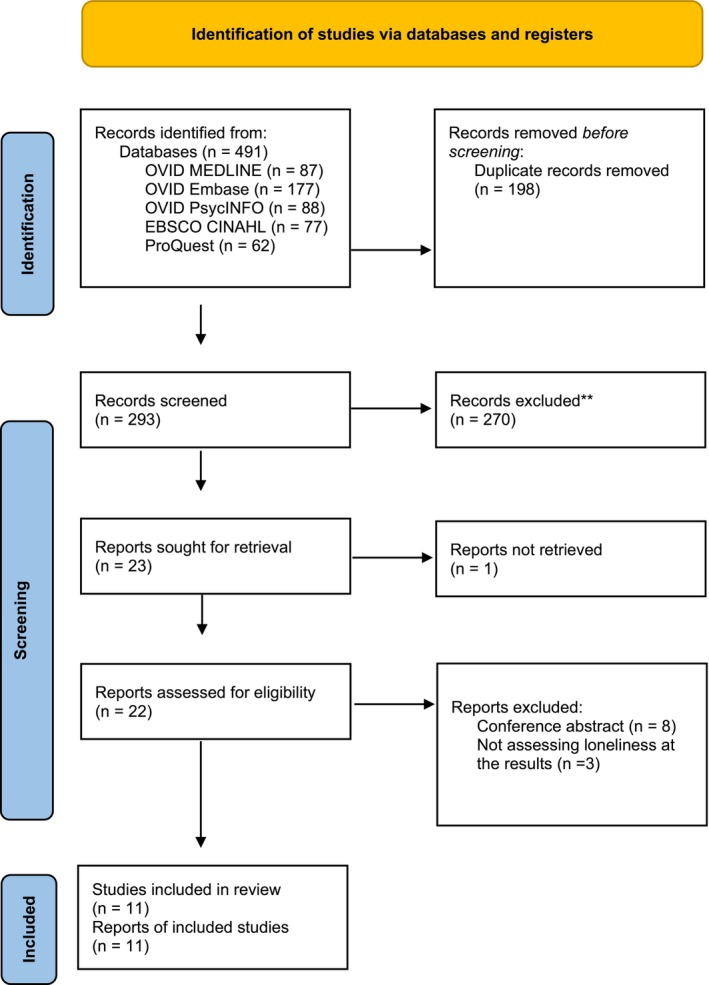
PRISMA flow diagram.

The search results were exported to EndNote (Version 20.6; available at https://endnote.com/) to remove duplicates. All citations were further imported to the Covidence review manager (available at www.covidence.com) before reviewing and screening. Two reviewers (L.Y.C. and Y.Z.) independently screened the title, abstract and full texts of articles for inclusion in the review. Conflicts between the two reviewers were discussed and solved by the third reviewer (K.H.M.H.), who is experienced in scoping review and loneliness. Figure 1 shows the PRISMA flow diagram of identifying and selecting studies.

### 4.5 Quality Appraisal

Although scoping review does not require a quality appraisal, we critically appraised the quality of included studies using JBI and mixed methods appraisal tools (MMAT) (Hong et al. 2018; Lockwood et al. 2015; Moola et al. 2020; Tufanaru et al. 2020). The results of the quality appraisal were presented in Tables 2, 3, 4. Quality appraisal further provides a comprehensive view of the current state of the art of the evidence. Following the suggestion of Arksey and O'Malley (2005), we did not exclude any articles because of quality issues.

**TABLE 2:** Critical appraisal using JBI (Quantitative study).

First Author (year)	Study design	Q1	Q2	Q3	Q4	Q5	Q6	Q7	Q8	Q9	Q10	Q11	Q12	Q13	No. of yes/total no. of questions
Fields et al. (2021)	Cross‐sectional study	Yes	Yes	Yes	Yes	Yes	No	Yes	Yes	Yes	Yes	NA	NA	NA	9/10
Franse et al. (2018)	Quasi‐experimental study	Yes	Yes	Yes	Yes	Yes	Yes	Yes	Yes	Yes	NA	NA	NA	NA	9/9
Jingna et al. (2012)	RCT	Yes	Yes	Yes	Unclear	Unclear	Unclear	Yes	Yes	No	Yes	Yes	Yes	Yes	9/13
Joosten‐Hagye et al. (2020)	Quasi‐experimental study	Yes	Unclear	Unclear	No	Yes	No	Yes	No	No	NA	NA	NA	NA	3/9
Keller et al. (2021)	Quasi‐experimental design	Yes	Yes	Yes	Yes	Yes	Yes	Yes	Yes	Yes	NA	NA	NA	NA	9/9
Rice et al. (2020)	Quasi‐experimental design	Yes	No	Yes	No	Yes	Yes	Yes	Yes	Yes	NA	NA	NA	NA	7/9
Richmond‐Cullen (2018)	Quasi‐experimental design	Yes	Unclear	Unclear	No	Yes	Yes	Yes	Yes	Yes	NA	NA	NA	NA	6/9
Van Lieshout et al. (2018)	RCT	Yes	Yes	Unclear	Yes	Unclear	Yes	Yes	Yes	Yes	Yes	Yes	Yes	Yes	11/13

**TABLE 3:** Critical appraisal using JBI (Qualitative study).

First Author (year)	Study design	Q1	Q2	Q3	Q4	Q5	Q6	Q7	Q8	Q9	Q10	No. of yes/total no. of questions
Chana et al. (2016)	Qualitative study	Yes	Yes	Yes	Yes	Yes	Yes	Yes	Yes	Yes	Yes	10/10
Meuser et al. (2022)	Qualitative study	Yes	Yes	Yes	Yes	Yes	Unclear	Unclear	Yes	No	Yes	7/10

**TABLE 4:** Critical appraisal using MAAT.

First Author (year)	Study design	S1	S2	1.1	1.2	1.3	1.4	1.5	3.1	3.2	3.3	3.4	3.5	5.1	5.2	5.3	5.4	5.5	No. of yes/total No. of questions
Beausoleil et al. (2024)	Mix‐method	Yes	Yes	Yes	No	Yes	Yes	Yes	Yes	No	Yes	Can't tell	Yes	Yes	Yes	Yes	Yes	Yes	14/17

### 4.6 Data Extraction

General characteristics of the included studies (e.g., author, year of publication, country of study, objectives, study design, settings, population characteristics and sample size) were extracted as shown in Table 5. Data relevant to the research questions (content and dosage, type of collaboration, involved discipline, mode of delivery, outcome or experience, facilitators and barriers) were extracted and are presented in Table 6. The data extraction was conducted by one reviewer, and the findings were cross‐checked by other members of the team.

**TABLE 5:** Characteristics of included studies.

Author (year)	Country of study	Objectives	Study design	Settings	Population characteristics	Measurement/indicators of loneliness	Sample size
Beausoleil et al. (2024)	US	To evaluate older adult volunteers' experience in a service‐learning programme in relation to social connectedness	Mix‐method study (qualitative group discussions via zoom and questionnaire survey)	Community	Older adult volunteers Mean age: 73.8 (range: 61–89) years Female: 72.5%	Five questions about social interactions and feelings of social connectedness	55
Chana et al. (2016)	England	To understand the attitude and perspective of intermediate care team professionals regarding loneliness	Qualitative study (semi‐structured individual interview)	Community	Intermediate care team professionals working with adults aged 60 years and above presenting with complex health and social care needs Mean age: Unclear Working experience: a minimum of 7 years working in intermediate care teams (range 7–11 years)	Participants' perceptions and experiences of loneliness	8
Fields et al. (2021)	US	To improve the psychological well‐being of older adults with and without cognitive problems	Quantitative study (Exploratory pilot uncontrolled pre‐test‐post‐test study)	Residential care setting	Older adults with and without cognitive impairment Mean age: 85.8 (SD 4.5) years Female: 73.3%	UCLA loneliness scale	15
Franse et al. (2018)	United Kingdom, Greece, Croatia, the Netherlands and Spain	To evaluate effectiveness of preventive care intervention in community‐dwelling older adults regarding their lifestyle, health and quality of life	Quantitative study (International multi‐centre pre‐post controlled trial)	Community	Older adults aged 75 years or older Mean age: 79.5 (SD 5.6) years Female: 60.8%	Jong Gierveld loneliness scale	1844
Jingna et al. (2012)	China	To assess the effectiveness of nurse‐led multidisciplinary team home visiting in the psychological well‐being of older adults living home	Quantitative study (randomised controlled trial)	Community	Older adults aged 80 or above with clear cognition Mean age: 84.76 (SD 3.90) years Female: 77.8%	UCLA loneliness scale	144
Joosten‐Hagye et al. (2020)	US	To alleviate loneliness and social isolation of older adults	Quantitative study (uncontrolled pre‐test‐post‐test study)	Community	Interprofessional graduate and doctoral students Older adults Mean age: Unclear Female: Unclear	One item asking about “my partner has benefited from social interaction”	115 (students)
Keller et al. (2021)	Germany	To assess the effectiveness of digital trainings for rehabilitation patients. To examine the relationship between communication with rehabilitation satisfaction and subsequently affect perceived rehabilitation success.	Quantitative study (controlled pre‐test‐post‐test study)	Psychosomatic rehabilitation clinic	Patients from psychosomatic rehabilitation clinics Mean age: Unclear Female: 64.4%	Two items: ‘How often do you feel lonely?’ stemming from the Center for Epidemiologic Studies–Depression (CES–D) Scale and ‘How often do you feel unhappy to be alone?’ from the UCLA Loneliness Scale	724
Meuser et al. (2022)	US	To involve older adults in internet communication To offer students practical experience	Qualitative study	Community	Students Older adults Mean age: Unclear Female: Unclear	Older adults' qualitative reflections on connections	54 (student) Approximately 250 (older adults)
Rice et al. (2020)	Australia	To evaluate the acceptability, feasibility and safety of a digital intervention targeting young people with social anxiety To examine the association between the intervention, clinical and social variables related to social anxiety	Quantitative study (uncontrolled pre‐test‐post‐test pilot study)	Community	Young people with social anxiety Mean age: 19.8 (SD 3.3) years Female: 51.7%	Revised UCLA Loneliness Scale	89
Richmond‐Cullen (2018)	US	To determine whether artists in residence programs will have a positive impact on the self‐reported loneliness of senior citizens	Quantitative study (uncontrolled pre‐test‐post‐test study)	Community	Senior citizens aged 60 years or older of the Commonwealth of Pennsylvania Mean age: Unclear Female: 70.4%	Revised UCLA Loneliness Scale	71
Van Lieshout et al. (2018)	The Netherlands	To examine the effectiveness of interdisciplinary and multicomponent intervention in preventing disability of older adults	Quantitative study (Randomised controlled trial)	Community	Pre‐frail older adults aged 65 or above Mean age: 74 (SD 7.2) years Female: 55.2%	Jong Gierveld Loneliness Scale	290

**TABLE 6:** Characteristics of IPC.

Author, year	Content and dosage	Duration	Multi/inter/trans‐professional/others	Involved discipline	Mode of delivery	Outcomes/experiences	Facilitators and barrier
Beausoleil et al. (2024)	Internet communication happened on consecutive Mondays, each discussion lasted for 75 min	4 weeks	Interprofessional	A variety of health care professions (major not specified)	Zoom	Participants' qualitative feedback: Being encouraged to find more social activities, reducing the feelings of social isolation Having a sense of purpose in sharing their stories with students Having genuine engagement in the program Participants' quantitative feedback: Participation in the programme had a positive effect on their mood (86%) and made them feel more socially connected (71%)	NA
Chana et al. (2016)	Not described	Not applicable	Intermediate care team	Nurse, occupational therapist and physiotherapist	Face to face	Not applicable	Facilitators Enhancing staff training Fostering stronger working relationships between the healthcare sector and other sectors Barriers Loneliness is less prioritised in service The service provided may not suit the need of the patient or not something they would tend to do. The negative attitude of the patients toward meeting new people Limited resources and increased workload will restrain health care professionals from reviewing complex or long‐term issue of the patients More consistent communication was needed for sharing understanding of the pressure of each component, improve relationship between co‐workers and further ensure the patients can get timely care
Fields et al. (2021)	Interactive sessions with a social robot	Three 10‐min sessions	Interdisciplinary	Theatre arts, social work, and engineering	Face‐to‐face	Significantly decrease in depression (from 2.51 ± 2.15 to 1.03 ± 0.66, *F* = 5.11, *p* = 0.008) and face scores (from 2.19 ± 1.08 to 1.87 ± 1.06, *F* = 2.72, *p* = 0.03) over six time periods A marginally significant decrease in loneliness (from 4.12 ± 0.92 to 3.44 ± 0.91, *F* = 2.42, *p* = 0.08) In participants	NA
Franse et al. (2018)	Group training for strength and balance, medication review/self‐managed medication, adherence app and social groups	Not described	Multidisciplinary	Medicine, nursing, pharmacy, social work, psychology, occupational therapy and physiotherapy	Face to face	Positive impact on loneliness (B = −0.18, 95% CI = −0.35 to −0.02, *p* = 0.033). Compared to the whole intervention group, adjusted significant effect were greater in terms of recurrent fall (OR = 0.58, 95% CI = 0.40–0.85), frailty (B = −0.44, 95% CI = −0.71 to −0.17) and physical health‐related quality of life (B = 1.22, 95% CI = 0.24–2.21)	NA
Jingna et al. (2012)	Home visiting	6 months, visiting took place once a week for first 3 months and once per 2 weeks for the last 3 months	Multidisciplinary	Medicine, nursing, psychology	Face to face	Loneliness (*p* < 0.001) and depression (*p* < 0.001) decreased significantly	NA
Joosten‐Hagye et al. (2020)	Phone call to older adults by students for 30 to 60 min twice a week	6 weeks	Interprofessional	Medicine, Physician Assistant, Social Work, Psychology, Physiotherapy, Occupational Therapy, Pharmacy, Dentistry and Public Health	Phone call	Students believed their served older partner have benefited from social interaction (*t* = −3.46, *p* = 0.0008) Perceived by students, they believed themselves have benefited from the social interaction (*t* = 3.94, *p* = 0.0002) Student believed they have built new friendship with older adults (*t* = 3.64, *p* = 0.0004)	NA
Keller et al. (2021)	Psychotherapy and skills training relating to reintegration and return to work	12 weeks	Interdisciplinary and multidisciplinary	Psychotherapy, physiotherapy and occupational therapy	Face to face	Results indicated a significant main effect across time for perceived loneliness (*F* (1662) = 4.00, *p* < 0.05)	NA
Meuser et al. (2022)	Telephone support and zoom sessions of forming peer support group, crafting, topical discussions, art and special lecture	8 weeks	Interprofessional	Social work, medicine and occupational therapy	Zoom and phone call	Older adult participants found the story sharing in the “peer connection support group” to be “a really good experience” Older adult participants reported that the programme allowed them to try new technology	NA
Rice et al. (2020)	Delivered therapy content through bespoke therapy comics, provided guidance and scheduled contact with clinical moderators	12 weeks	Multidisciplinary	Mental health clinicians, researcher, fiction writer and comic artist	Novel digital platform	Significant improvement of loneliness measured by UCLA (*d* = 0.63, *p* < 0.001, RCI: 39.13% improved). Improvement in social connectedness as measured by the Social Connectedness Scale. An effect size of *d* = 0.63 (*p* < 0.001) was reported. Significant improvement in social anxiety symptoms measured by Leibowitz Social Anxiety Scale. An effect size of d = 0.73 (*p* < 0.001) Decrease in depression symptoms and suicidality (PHQ‐9 full scale: *d* = 0.66, *p* < 0.001; suicidality item: *d* = 0.27, *p* = 0.026)	NA
Richmond‐Cullen (2018)	Artists offered 10 sessions (2 h each) in creating and examining art in different arm forms	10 weeks	Not indicated	Performing art, literature, visual art and folk art	Face to face	Less feeling of loneliness was reported by participants (*p* = 0.17). The mean difference between the pre‐test and post‐test was 1.380, reflecting that there was a decrease in participants' self‐reported loneliness by 1.38 points following participation in the artist in residence program under the measurement of Revised UCLA Scale of Loneliness	NA
Van Lieshout et al. (2018)	Medication review, sessions involving information on healthy diet, daily activities and social skills training	23 weeks	Interdisciplinary	Physiotherapy, nursing, food nutrition, welfare, pharmacy	Face to face	Less feelings of loneliness measured with the Jong Gierveld Loneliness Scale was reported but it was not significant (*p* = 0.18, *z* = −0.99) Less feelings of depression measured with the Hospital Anxious Depression Scale were reported but it was not significant (*p* = 0.35, *z* = −0.98)	NA

### 4.7 Synthesis

The PAGER framework (Bradbury‐Jones et al. 2022) was employed to provide a comprehensive analysis of the results. The framework consists of five domains: patterns, advances, gaps, evidence for practice and research recommendations. A patterning chart was created to give a summary of synthesised findings in Table 7.

**TABLE 7:** PAGER framework.

Pattern	Advances	Gaps	Evidence for practice	Research recommendations
Types and effects of the IPC	Incorporated more tele‐technology since 2020	Lack of translational or implementational studies to translate the benefit of IPC intervention (particularly those involved technology) into the real world. Lack of RCT for art‐based IPC intervention	Intergenerational approach with technology is feasible and beneficial for loneliness for both young and older adults. Art‐based intervention may be beneficial to folder adults	Conduct translational studies for IPC interventions employing technology or intergenerational approach. Conduct randomised controlled trial for art‐based IPC intervention
Types of collaboration and disciplines involved in IPC	In addition to health and social care professionals, artists and engineers are further involved in IPC	Lack of detail to differentiate multidisciplinary, interdisciplinary and interprofessional in practice. Multidisciplinary, interdisciplinary were used interchangeably. There was no IPC labelled as transdisciplinary. Nurses only involved in 4 studies out of 11 included studies	IPC is feasible in interventions tackling loneliness for both younger and older adults	In order to facilitate the translation of intervention into larger community, future studies can also provide more details about the collaborations and dynamics between disciplines. Participatory action research using a co‐design approach may be beneficial. Concept analysis can be conducted to differentiate multidisciplinary, interdisciplinary and interprofessional. Nursing can expand their role in IPC to tackle loneliness
Population groups served by IPC	Young adults emerged as a study population out from older adults.	There was only one study on younger adults. Most of the studies for older adults were conducted in community. Residential care home drew little attention	IPC studies on loneliness were traditionally focused on older adults	Future studies on IPC interventions for loneliness shall focus more on younger adults. IPC interventions for older adults in residential care homes can be conducted
Facilitators/barriers affecting IPC	NA	Information about facilitators and barriers to implement IPC intervention for loneliness was scant	NA	Qualitative studies or process evaluation shall be incorporated in implementation of IPC intervention for loneliness. Therefore, facilitators and barriers to implement IPC to tackle loneliness can be reported

## 5 Results

Four hundred and ninety‐one articles were identified by the electronic databases, of which 198 duplicates were removed. The title and abstract of 293 articles were screened and 270 articles were excluded. Twenty‐two full texts were screened and 11 articles were further excluded because of inappropriate study design and not assessing loneliness as an outcome. Finally, 11 articles were included in this review.

### 5.1 Characteristics and Quality of the Included Studies

Out of the 11 studies, eight were quantitative studies (Fields et al. 2021; Franse et al. 2018; Jingna et al. 2012; Joosten‐Hagye et al. 2020; Keller et al. 2021; Rice et al. 2020; Richmond‐Cullen 2018; Van Lieshout et al. 2018), two were qualitative studies (Chana et al. 2016; Meuser et al. 2022) and one was a mixed‐method study (Beausoleil et al. 2024). Regarding the geographical location of the studies, five studies were conducted in the United States (Beausoleil et al. 2024; Fields et al. 2021; Joosten‐Hagye et al. 2020; Meuser et al. 2022; Richmond‐Cullen 2018), one in the United Kingdom (Chana et al. 2016), one in Germany (Keller et al. 2021), one in China (Jingna et al. 2012), one in Australia (Rice et al. 2020), one in the Netherlands (Van Lieshout et al. 2018) and one involving five countries (the United Kingdom, Greece, Croatia, the Netherlands, Spain) (Franse et al. 2018). With respect to clinical location, 10 studies were conducted within the community setting (Beausoleil et al. 2024; Chana et al. 2016; Franse et al. 2018; Jingna et al. 2012; Joosten‐Hagye et al. 2020; Meuser et al. 2022; Rice et al. 2020; Richmond‐Cullen 2018; Van Lieshout et al. 2018), one in a residential care setting (Fields et al. 2021), and another in a psychosomatic rehabilitation clinic (Keller et al. 2021). The sample size of included studies varied from 8 to 54 in qualitative studies, and 15 to 1844 in quantitative studies. The mixed‐method study was conducted among 55 older adult volunteers.

Generally, most included studies demonstrated good quality. Out of 11 studies, eight studies scored ‘yes’ at a level of 70% or above (Beausoleil et al. 2024; Chana et al. 2016; Fields et al. 2021; Franse et al. 2018; Keller et al. 2021; Meuser et al. 2022; Rice et al. 2020; Van Lieshout et al. 2018), being regarded as good quality. Three studies scored ‘yes’ but at a level lower than 70%, showing relatively lower quality (Jingna et al. 2012; Joosten‐Hagye et al. 2020; Richmond‐Cullen 2018).

### 5.2 IPC Interventions and Their Effects on Loneliness

Interprofessional collaboration interventions for loneliness were generally delivered through (i) digital technology or telecommunication (Beausoleil et al. 2024; Keller et al. 2021; Meuser et al. 2022; Rice et al. 2020; Joosten‐Hagye et al. 2020) or (ii) face‐to‐face (Fields et al. 2021; Franse et al. 2018; Jingna et al. 2012; Richmond‐Cullen 2018; Van Lieshout et al. 2018). Most of the identified studies in the review were published within the last 5 years.

#### 5.2.1 Digital‐Technology or Tele‐Communication

Employing digital technology (e.g., Zoom, digital platform) or tele‐communication (e.g., telephone), either as a mode of delivery of therapy or intergenerational services, to address loneliness has become a recent trend for studies published in the recent 3 years. Meuser et al. (2022) conducted a telecollaboration in gerontology service learning for students from a variety of health‐related disciplines. The student facilitators were trained to provide 108 h of student‐led sessions via Zoom for 250 older adults. The interventional approaches included peer support, poetry appreciation, crafting, group reminiscence and art forums. Qualitative findings of older adults showed that the student‐led sessions were able to reconnect older adults and to offer some purposes for older adults during the idle period of the COVID‐19 pandemic.

Beausoleil et al. (2024) explored older adults' experience in a virtually formatted service learning programme provided by students from various health disciplines using a mixed methods design. The service learning programme involved four weekly 75‐min conversation modules between 112 undergraduate students and older adults. Older adults reported a positive effect on their mood and were more socially connected. Qualitative findings of older adults generally uncovered the benefits of reducing feelings of perceived social isolation, bringing joy, giving a sense of purpose and facilitating genuine engagement.

Keller et al. (2021) conducted a three‐arm controlled trial to investigate the effectiveness of digital training in reducing loneliness in 724 patients from an interdisciplinary psychosomatic rehabilitation clinic. There was a significant main effect across time for perceived loneliness in 724 patients from the psychosomatic rehabilitation clinic. The reduction in loneliness was observed specifically in the intervention groups that included enhanced digital group training components.

Rice et al. (2020) employed a multi‐disciplinary team to develop an online platform for delivering evidence‐based therapy for social anxiety via bespoke therapy comics, as well as a social network‐style platform to foster positive social connections and experiences with peer support. A 12‐week pilot study was conducted on 89 young people, who showed decreased loneliness.

Joosten‐Hagye et al. (2020) conducted an age‐friendly connection programme by having interprofessional students enrolled in graduate health‐related programmes pair up with older adults. Students engaged in 30‐ to 60‐min phone calls with older adults 2 to 5 times per week for 6 weeks. Students were prepared by faculty with asynchronous de‐briefing sessions via Zoom or email. Joosten‐Hagye et al. (2020) reported significant changes in student perceptions of (i) benefits on social interaction of older adults; (ii) benefits on students from having social interaction with older adults; and (iii) friendship between the students and older adults. However, Joosten‐Hagye et al. (2020) failed to report results from the perspective of older adults.

#### 5.2.2 Face‐To‐Face

Five studies employed a face‐to‐face mode of delivery for their IPC interventions, involving components of a befriending service, social support group, social skills training, home visiting or art‐related activity (Fields et al. 2021; Franse et al. 2018; Jingna et al. 2012; Richmond‐Cullen 2018; Van Lieshout et al. 2018).

Franse et al. (2018) conducted an international multi‐centre pre‐post controlled trial on the effectiveness of a coordinated care pathway for 1844 community‐dwelling older adults across five European cities. Major components for loneliness in the care pathway were mainly befriending services or social support groups run by volunteers or health/social care professionals. A positive effect was found on loneliness.

Van Lieshout et al. (2018) conducted a randomised controlled trial of an interdisciplinary multi‐component intervention on 290 pre‐frail older adults. The active component for loneliness was five weekly 2.5‐h social skills training sessions. However, it did not show significant effects on loneliness.

Jingna et al. (2012) conducted a randomised controlled trial to examine the impact of home visiting by a nurse‐led multi‐disciplinary team on loneliness among community‐dwelling older adults and found a significant decrease in loneliness at 3 and 6 months after intervention.

Two studies with art‐based components involving artists in the planning and implementation of the IPC intervention showed effects in reducing loneliness (Fields et al. 2021; Richmond‐Cullen 2018). Fields et al. (2021) conducted a pilot pre‐post study on 15 older adults with/without cognitive impairment from residential care homes. The intervention was three 10‐min participatory art sessions with interaction with social robots. The decrease in loneliness was significant for older adults with or without cognitive impairment. Another study involved 14 artists to provide 10 sessions in creating and critiquing art to 71 older adults in various art forms (performing arts, visual arts, and multi‐disciplinary/disciplinary arts). The loneliness of older adults showed a significant decrease after the intervention (Richmond‐Cullen 2018). However, none of the art‐based studies were randomised controlled trials.

Overall, a clear picture emerged that digital technology has been a focus for studies published from 2020 onwards. Meanwhile, other loneliness‐based interventions from social network strengthening to more complex coordinated multi‐component care pathways have emerged. The intergenerational approach in the delivery of intervention was also adopted by three studies (Beausoleil et al. 2024; Joosten‐Hagye et al. 2020; Meuser et al. 2022).

### 5.3 Types of Collaboration and Disciplines Involved in IPC

Nine of the included studies identified themselves as employing ‘multidisciplinary’ (Franse et al. 2018; Jingna et al. 2012; Rice et al. 2020), ‘interdisciplinary’ (Fields et al. 2021; Van Lieshout et al. 2018), ‘multidisciplinary and interdisciplinary’ (Keller et al. 2021) or ‘interprofessional’ approaches (Beausoleil et al. 2024; Joosten‐Hagye et al. 2020; Meuser et al. 2022). Another two did not explicitly label their model of collaboration (Chana et al. 2016; Richmond‐Cullen 2018). These studies involved various professionals from health and social care disciplines, including nurses, physicians, occupational therapists, physical therapists, psychologists, pharmacists and social workers (Franse et al. 2018; Jingna et al. 2012; Rice et al. 2020; Beausoleil et al. 2024; Joosten‐Hagye et al. 2020; Meuser et al. 2022; Van Lieshout et al. 2018). In addition to health and social care professionals, artists from various fields were also involved in some studies. These include collaborations between mental health professionals, comic artists, performing artists, artists in visual art and folk art (Rice et al. 2020; Richmond‐Cullen 2018). Furthermore, one study involved fiction writers and engineers (Fields et al. 2021). The process behind collaboration between these different professional groups varied across identified studies, examined in detail in the next section.

#### 5.3.1 Multi‐Disciplinary

To deliver the comprehensive care pathway for older adults, Franse et al. (2018) involved physiotherapists to provide group training for strength and balance; pharmacists to review medication; psychologists and social workers to facilitate social groups, buddying services by volunteers and trained assistants as care coordinators. The development of care plans for older adults was a shared decision between care coordinators, physicians, older adults and informal caregivers. A study of nurse‐led multi‐disciplinary home visits for older adults (Jingna et al. 2012) involved community nurses, general practitioners and psycho‐geriatricians. The role of general practitioners and psycho‐geriatricians was to provide training for home visits and to participate in case meetings for directing therapy. Community nurses provided regular home visits. Rice et al. (2020) developed a digital intervention platform with a multi‐disciplinary team composed of mental health clinicians, researchers, young adult fiction writers, a comic artist and young people with a lived experience of social anxiety. However, despite the multitude of collaborative activities, specific details about the process of collaboration between different professional disciplines to develop the digital interventions were not explicitly described.

#### 5.3.2 Interdisciplinary

An interdisciplinary team consisting of theatre arts, social work and engineering developed and implemented an intervention integrating theatre and social robots (Fields et al. 2021). However, a description of the ways of collaboration within the team to develop and integrate theatre arts and social robots is minimally disclosed. The interdisciplinary multi‐component intervention programme (Van Lieshout et al. 2018) involved a physiotherapist, a psychosocial nurse, a dietician and a professional in welfare to conduct medication reviews, sessions on promoting a healthy diet, daily activities and social skills training. While there were four components in the intervention, details about how each discipline was responsible for its own components contributing to a larger picture of the programme were unknown.

#### 5.3.3 Multi‐Disciplinary and Interdisciplinary

Keller et al. (2021) involved elements of psychotherapy, physiotherapy and occupational therapy to deliver digital training on rehabilitation goals and digital rehabilitation for patients from psychosomatic rehabilitation clinics. Keller et al. (2021) claimed that it was a multi‐disciplinary and interdisciplinary intervention. However, details about how various disciplines collaborated at multi‐disciplinary or interdisciplinary levels were not fully disclosed.

#### 5.3.4 Interprofessional

Three studies described interventions that were labelled as ‘interprofessional’ and involved Internet‐based communication methods such as telephone calls and Zoom meetings (Beausoleil et al. 2024; Joosten‐Hagye et al. 2020; Meuser et al. 2022). Regarding the collaborative context in which the interventions took place, these studies tested the effectiveness of their students' gerontology service‐learning programmes to enhance older adults' social connectedness. Interprofessional students from multiple disciplines, such as medicine, social work, psychology, physiotherapy and occupational therapy, have been involved in these programmes. Within the identified studies, there was a lack of information about how students from different professional disciplines worked together to develop shared goals or coordinated efforts, and how communication and decision‐making were facilitated among the team members.

#### 5.3.5 Without Explicit Label

Chana et al. (2016) examined the attitudes of nurses, occupational therapists and physiotherapists in an intermediate care team towards strategies for tackling loneliness amongst older adults. Their study examined the numerous barriers that exist for intermediate care teams to tackle loneliness together. Richmond‐Cullen (2018) studied collaboration between artists and staff in residential care homes in tackling loneliness. How the staff in residential care homes worked with artists to design and deliver the intervention was unknown. However, neither of them labelled their studies with any terms related to IPC (e.g., multi‐disciplinary, interdisciplinary) to address loneliness.

Overall, IPC intervention has drawn more attention in tackling loneliness, particularly in the recent 5 years. On top of the usual collaboration between health and social care professionals, artists and engineers were involved in IPC interventions to tackle loneliness. However, there was a general lack of details about how various disciplines came together to develop and implement interventions to tackle loneliness. The differentiation between multi‐disciplinary and interdisciplinary or interprofessional is blurred, or sometimes interchangeable.

### 5.4 Population Groups Served by IPC

Among the studies reviewed, only Rice et al. (2020) specifically focused on young individuals with social anxiety, with an average age of 19.8 ± 3.3 years. The majority of the other studies focused specifically on loneliness in older adults, with mean ages ranging from 73.8 to 85.8 years. One of them was conducted in rehabilitation settings (Keller et al. 2021), whilst the remaining eight were conducted in community settings.

### 5.5 Facilitators and Barriers Affecting the Implementation of IPC for Loneliness

Within the studies reviewed, only one study (Chana et al. 2016) investigated the facilitators and barriers to address loneliness by an intermediate healthcare team, including nurses, physiotherapists and occupational therapists. Facilitators included enhancing staff training and fostering stronger working relationships between disciplines. Loneliness was deemed to be a very relevant issue for intermediate care team clients.

However, loneliness was not a priority of commissioners who funded IPC services (Chana et al. 2016). It was also difficult to refer patients to appropriate professionals because of loneliness. Health and social care professionals also varied in their ability to identify and address loneliness (Chana et al. 2016).

## 6 Discussion

By employing the PAGER framework, this scoping review synthesised evidence on the use of IPC to address loneliness in various populations, especially among older adults in community settings. All the included studies were from 2018 to 2022, except for one study (Jingna et al. 2012). It showed IPC for tackling loneliness has drawn increasing attention in recent years. Generally, all the IPC interventions showed positive effects on loneliness, except Van Lieshout et al. (2018). In addition to health and social care professionals, students, volunteers, artists and engineers were also involved in IPC interventions for loneliness. Interventions were delivered through both face‐to‐face and tele‐technology. Apart from social support groups, social skill training, and home visiting, recent studies also involved more complex interventions, such as digital rehabilitation, coordinated care pathways, social robots and art‐based interventions.

In response to the Covid‐19 pandemic and the subsequent rise of loneliness (Priya Giri and Dubey 2023), researchers have increasingly employed digital technology or tele‐communication to deliver IPC interventions to tackle loneliness (Mumtaz et al. 2023). Three (Beausoleil et al. 2024; Joosten‐Hagye et al. 2020; Meuser et al. 2022) out of the five studies employing digital technology or tele‐communication involved undergraduate students as a modality of service learning to support older adults. On the other hand, Rice et al. (2020) delivered therapy through an online platform directly to young adults. This may imply the importance of taking an intergenerational approach when using digital technology to tackle loneliness among older adults. For example, Mariano et al. (2022) highlighted the under‐utilisation of digital technology in older adults, mainly due to the threat of ageist stereotypes. However, Freeman et al. (2020) showed that intergenerational support was able to promote technology acceptance among older adults because the younger generation can introduce and teach older adults how to use digital devices, computers and social networking sites. Therefore, while digital technologies offer convenience for the delivery of information and interpersonal connections, intergenerational relationships were suggested to offer an important lens to tackle loneliness among older adults (Ayalon and Segel‐Karpas 2024). However, as these studies were mainly service‐learning projects or randomised controlled trials, further information about the translation of findings into social care services is needed for the implementation of interventions with both intergenerational approach and digital technology.

For those IPC interventions that were provided through face‐to‐face or digital technologies, Van Lieshout et al. (2018) were the only study that did not report positive effects on symptoms of loneliness amongst older adults. Social skill training was the active component of IPC intervention for loneliness. According to the social–emotional selectivity theory (Löckenhoff and Carstensen 2004), older adults prioritised meaningful relationships over future‐oriented goals aimed at acquiring information and expanding horizons. As such, social skills training with minimal opportunities for meaningful interpersonal interactions with the wider community may have limited influence on loneliness among older adults. In accordance with a systematic review and meta‐analysis (Shekelle et al. 2024), it was deemed essential to incorporate activities that go beyond social skill‐building and provide individuals with meaningful opportunities for social interaction (e.g., social groups) in IPC interventions.

The scoping review identified a lack of comprehensive descriptions on the ways of collaboration between disciplines. Therefore, we are unable to provide sufficient details regarding professional dynamics, levels of sharing knowledge and their roles and responsibilities. However, shared decision‐making was shown to be promising in addressing mental health effectively (Pii et al. 2020). Therefore, providing details about the ways of various professionals committed to shared decision‐making can be beneficial for future IPC services. In addition, there was an issue that multi‐disciplinary, interdisciplinary and interprofessional were employed interchangeably in identified studies. Conceptual ambiguities can be a major obstacle to the advancement of psychological sciences (Bringmann et al. 2022). For example, we are unable to differentiate the levels of benefits of loneliness brought by various categories (e.g., multi‐disciplinary, interdisciplinary) of IPC. It will present difficulties for nurses or health and social care professionals to decide on the level of collaboration with various disciplines to achieve optimal outcomes on loneliness of various populations. To advance the field and provide practical guidance for implementation, future studies should aim to provide more comprehensive details on the dynamics of interprofessional collaborative activities in the management of loneliness. Participatory action research may be a possible direction to work out and describe the ways of collaboration. Furthermore, concept analyses on multi‐disciplinary, interdisciplinary, transdisciplinary and interprofessional are required.

Whilst a diverse range of health and social care professionals (e.g., medicine, social work, nursing, physiotherapy, occupational therapy) were involved, there were also studies involving non‐health disciplines (e.g., artists, fiction writers and engineers) (Fields et al. 2021; Rice et al. 2020). Increasing the involvement of more diverse non‐medical disciplines is encouraged, as loneliness is not a medical condition. It is more a psycho‐social‐spiritual condition that can result in negative health consequences (Ho et al. 2021; Ho, Chung, et al. 2023; Ho, Hung, et al. 2023; Ho, Yang, et al. 2023). Surprisingly, nursing professionals were only involved in four of the studies identified (Chana et al. 2016; Franse et al. 2018; Jingna et al. 2012; Van Lieshout et al. 2018). Given that gerontological nursing shoulders a great deal of the responsibility to promote health and wellbeing of older adults in the community, it is important for nursing professionals to expand their roles in IPC to help tackle psycho‐social‐spiritual problems for people with loneliness.

The scoping review revealed a limited focus on the facilitators and barriers of IPC interventions for loneliness, with only one study addressing this issue. Chana et al. (2016) suggested that enhancing staff training and fostering stronger working relationships would facilitate IPC service. However, the study highlighted the problem of a medical framework in the current health and social care system, in which loneliness was not a sound reason to make referrals to related professionals. Furthermore, medically oriented disciplines (e.g., nursing, physiotherapy, occupational therapy) perceived themselves as having little knowledge and capability to identify people at risk of loneliness and to tackle loneliness. The qualitative findings of Chana et al. (2016) were in accord with the World Health Organization (2021) that loneliness is a neglected social determinant of health. As such, it is important to include loneliness in nursing education, particularly for gerontological nursing, as well as education for other helping professionals.

## 7 Limitations

Most studies were about older adults in community settings, with only one study for young adults. As such, generalising the findings of this scoping review to other age groups should be done with caution. However, it also implies that loneliness among older adults is a prevalent problem to be tackled. Although we successfully identified a study in the Chinese language (Jingna et al. 2012), all the other recent studies were conducted in Western countries. Since loneliness involves psycho‐social‐spiritual aspects, it may be highly subject to cultural diversity. Studies other than Western countries are urgently needed.

## 8 Conclusion

This scoping review mapped and synthesised evidence on IPC for loneliness. IPC interventions for loneliness have become more popular since 2018. Employing digital technology was identified to be a recent trend in IPC for loneliness. Older adults have been a focus of IPC interventions for loneliness. However, the ways of collaborating between disciplines to produce IPC interventions were unclear. There is a strong need to differentiate the concepts of multi‐disciplinary, interdisciplinary, transdisciplinary and interprofessional. Around the world, nursing professionals should be encouraged to play an active role in IPC interventions for loneliness, expanding the influence of nursing to psycho‐social‐spiritual dimensions of human wellbeing. Finally, our findings highlight the importance of including loneliness‐related education, across the whole lifespan, within undergraduate nursing education and gerontological nursing.

## Author Contributions

**Lok Ying Chu:** conceptualisation, methodology, investigation, visualisation, writing – original draft. **Youjuan Zhang:** methodology, investigation, writing – reviewing and editing. **Graeme Drummond Smith:** writing – reviewing and editing. **Ken Hok Man Ho:** conceptualisation, methodology, investigation, visualisation, supervision, writing – original draft; writing – reviewing and editing.

## Ethics Statement

The authors have nothing to report.

## Conflicts of Interest

The authors declare no conflicts of interest.

## Supporting information


Appendix S1.

## Data Availability

The data that support the findings of this study are available from the corresponding author upon reasonable request.
