# Supplementary material for: Interprofessional Collaborations on Interventions for People With Loneliness: A Scoping Review
Source: Nurs Open. 2025 May 21;12(5):e70239. doi: 10.1002/nop2.70239 (PMC12093052; doi:10.1002/nop2.70239)
Supplement: Supplementary file 1 — Appendix S1. [file NOP2-12-e70239-s001.docx]

Search strategy for OVID MEDLINE dated 6-10-2023

| # | Searches | Searches results |
| --- | --- | --- |
| 1 | loneliness.tw. | 12220 |
| 2 | perceived loneliness.tw. | 201 |
| 3 | interprofessional collaboration.tw. | 2693 |
| 4 | interprofessional.tw. | 14807 |
| 5 | multidisciplinary.tw. | 118896 |
| 6 | multiprofessional.tw. | 1777 |
| 7 | transdisciplinary.tw. | 2756 |
| 8 | Transprofessional.tw. | 23 |
| 9 | Interdisciplinary.tw. | 48108 |
| 10 | interprofessional communication.tw. | 726 |
| 11 | Interdisciplinary relations.tw. | 33 |
| 12 | Interprofessional relations.tw. | 98 |
| 13 | 1 or 2 | 12220 |
| 14 | 3 or 4 or 5 or 6 or 7 or 8 or 9 or 10 or 11 or 12 | 181193 |
| 15 | 13 and 14 | 124 |
| 16 | limit 15 to (humans and yr="2003 - 2023") | 87 |

Search strategy for OVID Embase dated 17-02-2025

| # | Searches | Searches results |
| --- | --- | --- |
| 1 | loneliness.tw. | 17196 |
| 2 | perceived loneliness.tw. | 254 |
| 3 | interprofessional collaboration.tw. | 3763 |
| 4 | interprofessional.tw. | 20622 |
| 5 | multidisciplinary.tw. | 218168 |
| 6 | multiprofessional.tw. | 3096 |
| 7 | transdisciplinary.tw. | 3692 |
| 8 | Transprofessional.tw. | 37 |
| 9 | Interdisciplinary.tw. | 75665 |
| 10 | interprofessional communication.tw. | 1088 |
| 11 | Interdisciplinary relations.tw. | 39 |
| 12 | Interprofessional relations.tw. | 107 |
| 13 | 1 or 2 | 17196 |
| 14 | 3 or 4 or 5 or 6 or 7 or 8 or 9 or 10 or 11 or 12 | 311757 |
| 15 | 13 and 14 | 232 |
| 16 | limit 15 to (humans and yr="2003 - 2023") | 193 |

Search strategy for OVID PsycINFO dated 17-02-2025

| # | Searches | Searches results |
| --- | --- | --- |
| 1 | loneliness.tw. | 11831 |
| 2 | perceived loneliness.tw. | 217 |
| 3 | interprofessional collaboration.tw. | 1490 |
| 4 | interprofessional.tw. | 6170 |
| 5 | multidisciplinary.tw. | 25021 |
| 6 | multiprofessional.tw. | 561 |
| 7 | transdisciplinary.tw. | 2409 |
| 8 | Transprofessional.tw. | 11 |
| 9 | Interdisciplinary.tw. | 30521 |
| 10 | interprofessional communication.tw. | 264 |
| 11 | Interdisciplinary relations.tw. | 39 |
| 12 | Interprofessional relations.tw. | 143 |
| 13 | 1 or 2 | 16328 |
| 14 | 3 or 4 or 5 or 6 or 7 or 8 or 9 or 10 or 11 or 12 | 62060 |
| 15 | 13 and 14 | 133 |
| 16 | limit 15 to (humans and yr="2003 - 2023") | 97 |

Search strategy for EBSCO CINAHL dated 6-10-2023

| # | Searches | Searches results |
| --- | --- | --- |
| 1 | AB loneliness or AB perceived loneliness | 7017 |
| 2 | AB ( interprofessional collaboration or interprofessional teamwork or multidisciplinary ) OR interprofessional OR 9multidisciplinary team or integrative team or interdisciplinary team or interprofessional team ) OR transdisciplinary OR transprofessional | 119181 |
| 3 | 1 and 2 | 77 |

Search strategy for ProQuest dated 17-2-2025

| # | Searches | Searches results |
| --- | --- | --- |
| 1 | abstract(loneliness) AND abstract(interprofess* OR multidisciplin* OR interdiciplin* OR transprofess* OR transdisciplin* OR multiprofess*) | 88 |
